# Combined structural analysis and cathodoluminescence investigations of single Pr^3+^-doped Ca_2_Nb_3_O_10_ nanosheets

**DOI:** 10.1038/s41598-023-35142-3

**Published:** 2023-05-17

**Authors:** Rasa Changizi, Stefan Zaefferer, Christian Ziegler, Vitaliy Romaka, Bettina V. Lotsch, Christina Scheu

**Affiliations:** 1 Max-Planck-Institut Für Eisenforschung GmbH, Max-Planck-Straße 1, 40237 Düsseldorf, Germany; 2 Department of Chemistry, University of Munich (LMU), Butenandtstraße 5-13, 81377 München, Germany; 3 Leibniz Institute for Solid State and Materials Research (IFW) Dresden, Helmholtzstr. 20, 01069 Dresden, Germany; 4 Max Planck Institute for Solid State Research, Heisenbergstraße 1, 70569 Stuttgart, Germany

**Keywords:** Materials science, Nanoscience and technology, Optics and photonics

## Abstract

Due to the novel properties of both 2D materials and rare-earth elements, developing 2D rare-earth nanomaterials has a growing interest in research. To produce the most efficient rare-earth nanosheets, it is essential to find out the correlation between chemical composition, atomic structure and luminescent properties of individual sheets. In this study, 2D nanosheets exfoliated from Pr^3+^-doped KCa_2_Nb_3_O_10_ particles with different Pr concentrations were investigated. Energy dispersive X-ray spectroscopy analysis indicates that the nanosheets contain Ca, Nb and O and a varying Pr content between 0.9 and 1.8 at%. K was completely removed after exfoliation. The crystal structure is monoclinic as in the bulk. The thinnest nanosheets are 3 nm corresponding to one triple perovskite-type layer with Nb on the B sites and Ca on the A sites, surrounded by charge compensating TBA^+^ molecules. Thicker nanosheets of 12 nm thickness (and above) were observed too by transmission electron microscopy with the same chemical composition. This indicates that several perovskite-type triple layers remain stacked similar to the bulk. Luminescent properties of individual 2D nanosheets were studied using a cathodoluminescence spectrometer revealing additional transitions in the visible region in comparison to the spectra of different bulk phases.

## Introduction

Atomically thin nanosheets, termed “two-dimensional” materials, have been lately a topic of interest^1,2^. This is due to the novel properties related to the quantum confinement effects and the large number of active surface atoms relative to the bulk. Butler et al.^3^ defines two-dimensional (2D) material as a material in which the atomic arrangement and bond strength along two-dimensions are similar and much stronger than along the third dimension^3^. The fact that 2D materials have different properties compared to the bulk^1^ has attracted attention in the scientific community. Unique chemical and physical properties, improved mechanical flexibility, enhanced electrical and optoelectronic properties, among others, are mentioned in the literature^1,3–5^. Graphene is an archetypical 2D material which was prepared in 2004 by Geim’s group through the Scotch tape exfoliation of graphite^6^.

There is a growing interest in 2D rare-earth nanomaterials because of the novel luminescent and magnetic properties offered by 4f–4f transitions^1^. Due to the inner 4f–4f energy level transitions, lanthanide ions can emit photons with almost the same energy while being surrounded by different chemical environments^4^. Even though luminescence of lanthanide ions doped into 2D nanosheets is rarely studied^7^, the existing literature related to the topic is briefly summarized here. As the focus is on the luminescence properties, recent developments on synthesis approaches are not included here. Liu et al.^4^ found that doping lanthanide ions into 2D nanosheets can adjust the emission region. Their photoluminescence (PL) measurements have shown that doping Nd^3+^ ions broadens the emission region of In_2_Se_3_ nanosheets from the visible to the near infrared (NIR) range. According to this group^4^, 2D In_2_Se_3_:Nd^3+^ nanosheets with strong NIR luminescence can be used in NIR photonic nanodevices and biomedical applications. The PL emission of lanthanide-containing NaYF_4_ 2D nanosheets was investigated by Clarke et al^5^. They mentioned that exfoliated 2D materials commonly exhibit a bandgap widening after exfoliation, while their NaYF_4_:Yb, Er nanosheets display a noticeable narrowing of the bandgap. This was confirmed by density functional theory (DFT) calculations and valence band photoemission measurements^5^. They reported that by decreasing the nanosheet thickness, the PL emission becomes weaker, which is due to the presence of fewer excited ions^5^. In another study, Bai et al.^8^ observed that the introduction of lanthanide ions can expand the intrinsic narrow-band emission of 2D layered transition metal dichalcogenides (TMDs) as determined by PL data. To prove their statement, they doped Er^3+^ into the lattice of bi-layered MoS_2_ and found that NIR emission was indeed obtained. Huang et al.^7^ developed a strategy to enhance the luminescence properties of MgWO_4_:Ln^3+^ (Ln = Eu, Tb) nanosheets through incorporation of carbon dots (CDs) to form CDs@ MgWO_4_:Ln^3+^ nanostructures. They reported that the incorporation of CDs with MgWO_4_:Ln^3+^ nanosheets had a slight effect on the morphology and phase structure but increased the PL emission intensity of CDs@ MgWO_4_:Eu^3+^ and CDs@MgWO_4_:Tb^3+^ nanosheets. They claimed that the luminescence enhancement mechanism was due to the capture of electrons by CDs and energy transfer between CDs and luminescent Ln^3+^. These CDs have a size of 3–5 nm ^7^. In a recent study, Awaya et al.^9^ reported changes in the PL properties for Eu^3+^/Tb^3+^-sandwiched TiNbO_5_ nanosheets under different pH conditions. Emission lines are observed at 545 nm (^5^D_4_ → ^7^F_5_ transition of Tb^3+^) and 614 nm (^5^D_0_ → ^7^F_2_ transition of Eu^3+^) for low and high pH, respectively.

In the present work, we study the luminescence behavior of Pr^3+^-doped Ca_2_Nb_3_O_10_ nanosheets on a local scale using a cathodoluminescence (CL) setup in the scanning electron microscope (SEM). The optical properties of individual nanosheets with different thicknesses are correlated to their crystal structure and chemical composition obtained via transmission electron microscopy (TEM) and energy dispersive X-ray spectroscopy (EDX). We found that due to the lower Pr content observed in the nanosheets compared to bulk particles^10,11^, different luminescence properties are observed.

## Experimental procedures

To obtain 2D nanosheets, Pr^3+^-doped KCa_2_Nb_3_O_10_ powder particles with a nominal formula K_1__−x_Ca_2-x_Pr_x_Nb_3_O_10_, [x = 0.05 and 0.50] were exfoliated in a 2-step process. First, K was exchanged against protons and subsequently exchanged against a bulky organic cation such as tetra-*n*-butylammonium (TBA^+^). As a result, individual Pr^3+^-doped [Ca_2_Nb_3_O_10_]^−^ nanosheets were produced. More details can be found in^12^.

The crystal structure of individual sheets was investigated by high-resolution transmission electron microscopy (HRTEM) imaging and electron diffraction. The experiments were performed in a Thermo Fisher Scientific Titan Themis TEM which has an X-FEG and an aberration corrector for the objective lens. An acceleration voltage of 300 kV was used to operate the microscope. A second Thermo Fisher Scientific Titan Themis STEM equipped with a C_s_ probe corrector was used for high angle annular dark-field (HAADF) imaging. A convergence angle of 23.8 mrad was used for the probe, with a spot size of around 1 Å. The operation voltage was 300 kV. To determine the chemical composition of the individual sheets a super X-detector from Bruker was used to perform EDX analysis. Acquisition time for each EDX map was 25 min to obtain good statistics with a high signal to noise ratio. TEM samples of the nanosheets were prepared by mixing the sheets into ethanol and deionized water. To increase the dispersion of the sheets, the solution was placed in an ultrasonicator for 20 min. The final suspension was dropped onto a TEM grid (Cu or Au) covered by an amorphous holey-carbon film and dried.

HRTEM images were analyzed and interpreted based on simulations using the Java Electron Microscopy Simulation (JEMS) software^13^ for a pseudo 2D crystal structure of [Ca_2_Nb_3_O_10_]^−^ nanosheets. For this, a CIF file was created as follows. To construct a set of 2D nanosheets and represent it as a 3D periodic structure, several necessary structural transformations were performed, including origin shift, supercell construction, K atom removal, and vacuum slab creation, using the VESTA program^14^. At the first stage, the origin of the initial crystal structure was changed by 0.5 along the c basis vector to position all atoms of the 2D sheet in the central part of the unit cell. After that, a 1 × 1 × 2 supercell was created by changing the value of the *P*_33_ element of the transformation matrix (*P*) from 1 to 2. To be able to freely remove individual atoms for the creation of the vacuum slab the symmetry of the structure was removed (reduced to space group *P*1) while keeping the atomic coordinates of all generated atoms in the supercell unchanged. The thickness of the slab is 1.4–1.5 nm. At the final stage, all K atoms were removed from the structure, as well as all atoms with *z*-coordinate ≥ 0.5. After these structural transformations, half of the unit cell along the *c*-direction contains the created 2D nanosheet which is separated from the same nanosheet in the neighboring unit cell by the vacuum slab, so that the interaction between the 2D sheets is minimized. The resulting structure was exported to CIF for image simulations.

A FEG SEM (Zeiss SEM450) equipped with DELMIC SPARC system was used to acquire the CL data of the nanosheets. The device has a motorized parabolic mirror which is used to reflect the generated light towards the lenses. Investigations were performed at an electron acceleration voltage of 10 kV, a beam current of 5.5 nA and a working distance of 14 mm. For the acquisition no sample cooling system was used. The CL data was acquired in the visible light wave length regime (450–650 nm). For CL investigation, the nanosheets were mixed with deionized water and dispersed on a Si substrate.

## Results and discussions

### EDX analysis of the nanosheets

**Figure 1:**
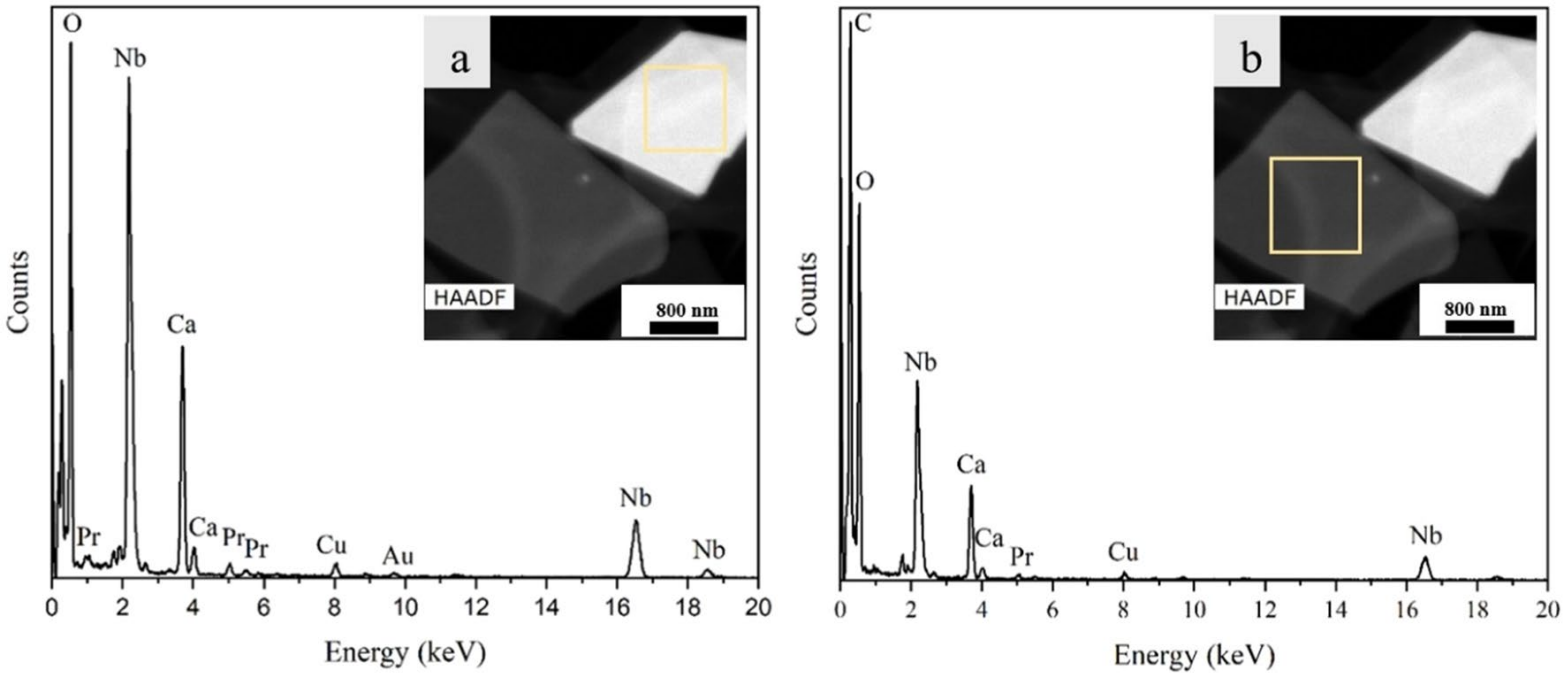
HAADF micrograph and corresponding EDX spectra of individual Pr^3+^-doped Ca_2_Nb_3_O_10_ nanosheets synthesized with a low Pr content: (**a**) thicker nanosheet and (**b**) thin nanosheet. The spectra were extracted from the regions marked with a yellow square.

EDX measurements were performed on 2D nanosheets exfoliated from K_1−x_Ca_2−x_Pr_x_Nb_3_O_10_, [x = 0.05 and 0.50]. The first group was supposed to have a low concentration of Pr and in the second group, a high Pr concentration. To prove this experimentally, EDX spectra were acquired on up to 30 nanosheets. For the 2D nanosheets with lower concentration of Pr used during the synthesis (x = 0.05), the HAADF image and corresponding EDX spectra are shown in Fig. 1. As seen in this figure, two individual nanosheets with different brightness in the HAADF image were investigated. The different brightness indicates a different thickness and /or different composition. The brighter appearing sheet in Fig. 1a shows a higher peak intensity for the Nb-K_α_, Nb-K_β_, Nb-L and Ca-K_α_ and Pr-L lines than the dark appearing sheet (Fig. 1b) which shows the same element specific lines. The quantitative evaluation of the EDX data reveals that the ratio between the elements for both sheets stays the same. This indicates that the difference in brightness in the HAADF image is related to a different thickness of the nanosheets but not a difference in composition. However, due to the absorption phenomena for light elements, the O ratio with respect to other elements is different for different sheets. As a result, the ratio of O and Nb changes between the two sheets. Based on the EDX data, the exfoliation process was successful, indicated by the absence of K. Additional peaks belong to C, and other contaminations such as Au and Cu which belong to the TEM grid and other artefacts.

**Figure 2:**
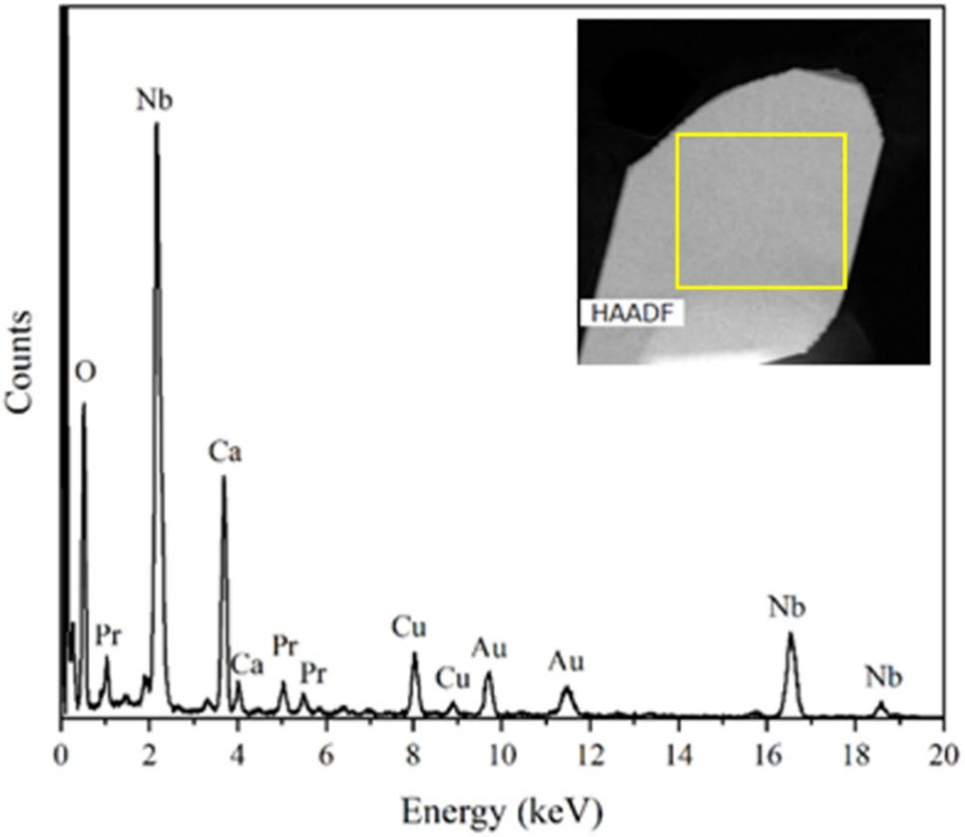
EDX spectrum of an individual Pr^3+^-doped Ca_2_Nb_3_O_10_ nanosheet synthesized with a higher Pr content and corresponding HAADF image where the area of data acquisition is marked.

Similar observations were made for the nanosheets which were synthesized with a higher concentration of Pr (x = 0.5). Figure 2 presents the HAADF image and the EDX spectrum of a single nanosheet from this sample. Again, no K was found but the main peaks show the presences of Ca, Nb, Pr and O, while the other impurity peaks are not related to the sheets.

EDX data sets of several individual nanosheets were taken, analysed and quantified to obtain the average elemental composition of the sheets. The results for fifteen nanosheets (ten with higher Pr content and five with lower Pr content according to the synthesis procedure) were averaged and are presented in Table 1. For single nanosheets with a higher Pr content (x = 0.5), the averaged atomic percentage were obtained as follows: 58.1 at% O, 14.5 at% Ca, 25.6 at% Nb and 1.8 at% Pr. Moreover, Ca and Nb over Pr ratios are calculated and averaged and also listed in Table 1. The same analysis was done for the single sheets with a low Pr (x = 0.05) content. The averaged results are given as: 52.0 at% O, 17.9 at% Ca, 27.2 at% Nb and 0.9 at% Pr. Comparing the average atomic percent for Ca and Nb for the different types of sheets revealed that a different number of Ca and Nb were substituted by Pr. The average Pr concentration for the high Pr content sheets (1.8 at%) is doubled compared to the Pr concentration for the lower Pr content sheets (0.9 at%).

**Table 1:** EDX measurements on several individual nanosheets.

Type of nanosheets	O_ave_ (at%)	Ca_ave_ (at%)	Nb_ave_ (at%)	Pr_ave_ (at%)	(Ca/Pr)_ave_	(Nb/Pr)_ave_
High Pr content	58.1	14.5	25.6	1.8	8.4	14.7
Standard deviation	11	3.5	7.1	0.5	0.9	1.1
Low Pr content	52.0	17.9	27.2	0.9	19.8	30.6
Standard deviation	13.1	4.8	5.9	0.2	1.1	4.1

In summary, based on the EDX results, we can conclude that the nanosheets synthesized with different Pr content indeed contained different amount of Pr, while nanosheets synthesized with the same Pr content had the same Pr atomic ratio with a low standard deviation (see Table 1) independent of the sheet thickness. Since there was no trace of K, the exfoliation process was successful. Average Pr concentration for the nanosheets synthesized with high Pr content is twice more than the nanosheets synthesized with low Pr content. However, the difference is much lower than the estimated one from the nominal precursor ratio. This indicates that part of the Pr cannot be incorporated in the lattice and that a solubility limit exists for the Pr^3+^-doped [Ca_2_Nb_3_O_10_]^−^ nanosheets. We did not systematically study the solubility limit but we observed that doping the same bulk KCa_2_Nb_3_O_10_ precursor with 10 at% Pr leads to a phase decomposition^10^. Two different phases with different crystal structures, namely PrNbO_4_ and Pr^3+^: Ca_2_Nb_2_O_7_ were found. To prevent this phase separation, a lower amount of Pr was doped in the bulk particles used for exfoliation [K_1-x_Ca_2-x_Pr_x_Nb_3_O_10_ with x = 0.05 and 0.50].

### Structural analysis of the nanosheets in (S)TEM

**Figure 3:**
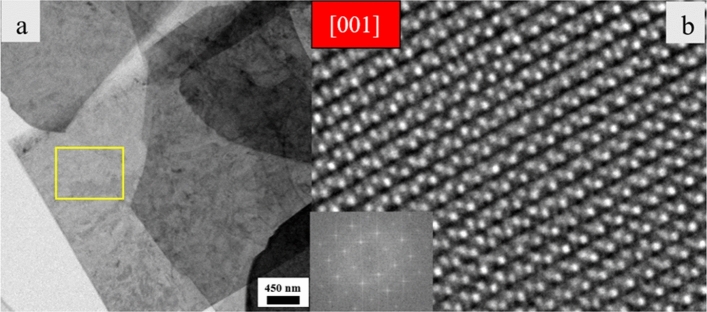
(**a**) Bright field TEM image of an assembly of 2D nanosheets. (**b**) HRTEM image of the marked area indicated in yellow in (**a**), showing a set of stacked nanosheets taken in [001] zone axis; the inset indicates the FFT pattern.

**Figure 4:**
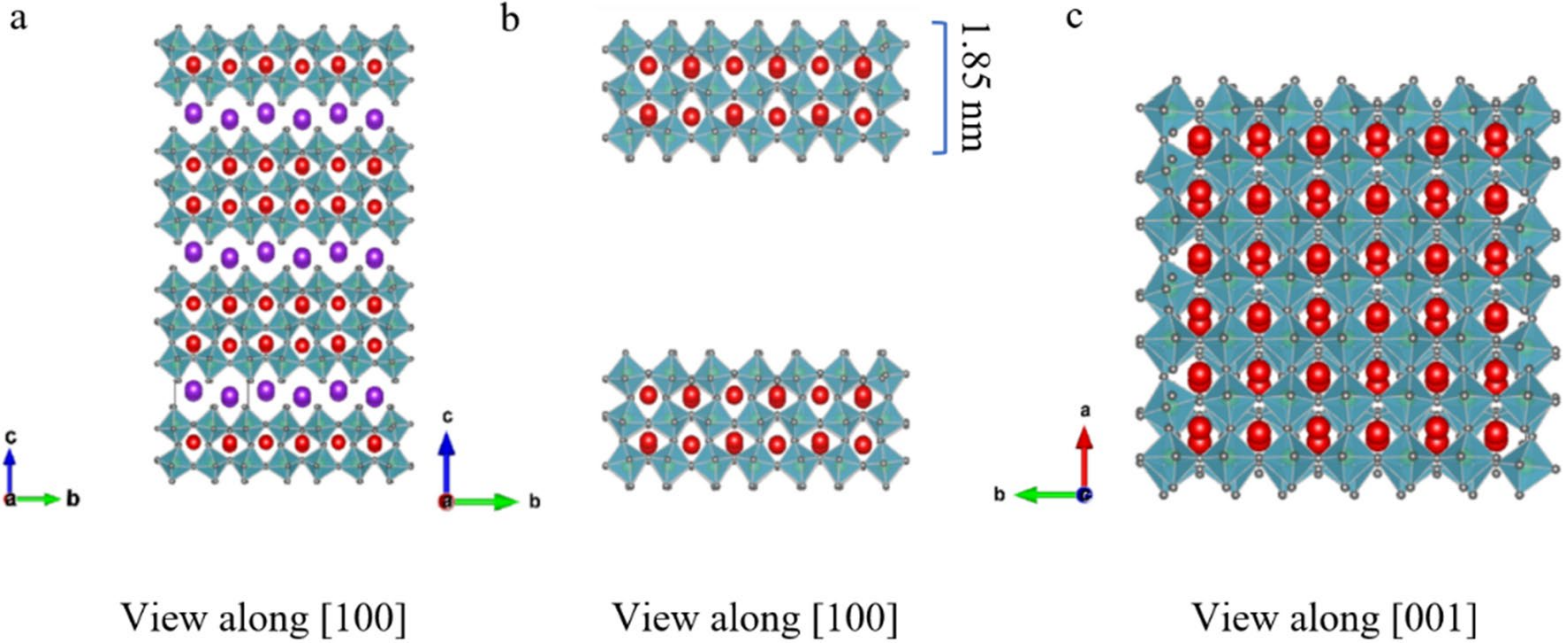
Crystal structure of (**a**) KCa_2_Nb_3_O_10_ viewed in [100] and pseudo 2D [Ca_2_Nb_3_O_10_]^−^ viewed in (**b**) [100] and (**c**) [001] (red spheres, Calcium; gray, Oxygen and purple, Potassium); Nb (shown with green spheres) is located within the centre of the octahedra.

Structural investigations of Pr^3+^-doped 2D nanosheets with lower Pr content were carried out in the C_s_-corrected (S)TEM. As shown in Fig. 3a, nanosheets tend to lie on top of each other and possess a lateral dimension of about 5 µm. To further study the atomic structure, one thin region (marked in yellow) was chosen. An HRTEM image of the selected area is illustrated in Fig. 3b. The region was oriented in [001] as determined by the Fast Fourier transform (FFT) pattern and by the electron diffraction pattern shown in Supplement S2. The FFT and electron diffraction pattern have no additional (rotated) reflections indicating that they belong to only one sheet with no other sheet below or on top of it. The crystal has a monoclinic structure like the bulk KCa_2_Nb_3_O_10_ precursor (ICSD 157839-see Fig. 4a) but with only three layers of connected Nb–O octahedra with Ca ions positioned in between and the K layers removed. In Fig. 4b and c, the pseudo 2D crystal structure of [Ca_2_Nb_3_O_10_]^−^ in [100] and [001] is viewed. A similar structure was observed for undoped nanosheets by Virdi et al.^15^ By using atomic force microscopy (AFM) measurements, they reported the thickness of a single nanosheet to be between 1.85 and 3 nm. The latter was due to the presence of TBA^+^ molecules on top of the surface ^15^. Moreover, Li. et al.^16^, observed a thickness of 1.85 nm for undoped nanosheet using AFM under vacuum conditions.

To obtain HRTEM images a negative C_s_ value was used. This condition gives enhanced contrast due to contributions of both amplitude and phase contrast^17^. According to^18^, an optimum contrast for samples up to 4 nm thickness is achieved if a negative C_s_ value with a positive defocus is used.

**Figure 5:**
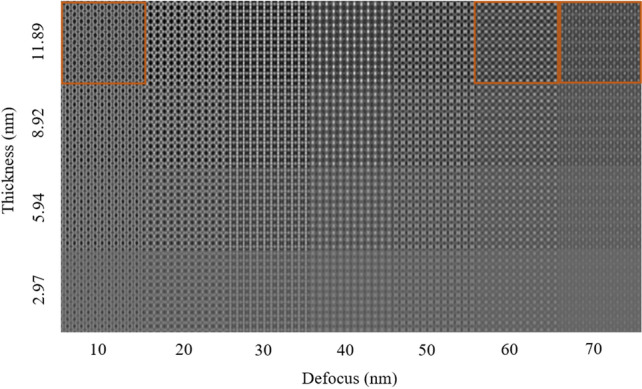
Defocus-thickness map of pseudo 2D [Ca_2_Nb_3_O_10_]^−^ nanosheet viewed along [001] and simulated with the multislice method. Accelerating voltage = 300 kV, C_s_ = − 0.01 mm.

Multislice simulations were carried out for the pseudo 2D structure of [Ca_2_Nb_3_O_10_]^−^ nanosheets along the [001] direction. The simulations were done to determine the thickness of the sheets and indicate the atomic positions of Nb, Ca and O. According to^10,11^, possible sites substituted by Pr could be Ca and Nb. The corresponding thickness-defocus map is shown in Fig. 5. In the simulated images, the thickness and the defocus values vary between 2.97–11.89 nm and 10–70 nm, respectively. The marked areas are compared with the HRTEM images taken at different defocus values. It is important to note that a larger thickness than 1.85 nm would indicate i) the presence of TBA^+^ molecules and ii) that some of the sheets remain stacked as in the bulk even after exfoliation.

**Figure 6:**
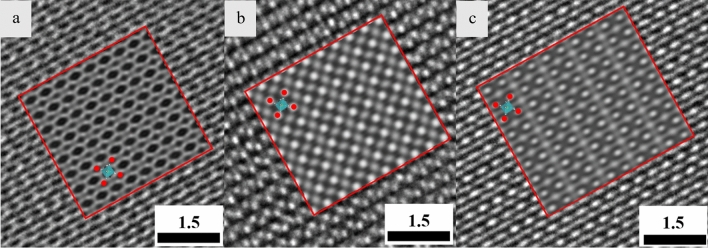
Experimental HRTEM images superimposed with simulated images of pseudo 2D [Ca_2_Nb_3_O_10_]^−^ nanosheet along [001] with a thickness value = 11.89 nm for (**a**) defocus value = 10 nm, (**b**) defocus value = 60 nm, and (**c**) defocus value = 70 nm. Simulated images are marked with red frames. Red, green and gray spheres indicate Calcium, Niobium and Oxygen, respectively. Nb is located inside the octahedra.

HRTEM images were taken at a thicker nanosheet for different defocus values. The results are compared with the simulated images and displayed in Fig. 6. As seen on this image, the simulated images (shown with a red frame) resemble the contrast of the experimental HRTEM images. The difference in the contrast we believe is related to the pseudo 2D structure defined in the CIF file (presence of vacuum slab). Moreover, astigmatism and small-angle mistilts in the experiment, as well as minor differences of defocus or spherical aberration values in the simulation should be mentioned. For the defocus value of 10 nm and thickness around 11.89 nm, the best fit was obtained. For this condition, Ca atoms are giving a bright contrast. On the other hand, Nb and O atoms appear dark. For the defocus value of 60–70 nm and the same thickness, all atoms Ca, Nb and O atoms show a bright contrast. Based on Fig. 6b, we conclude that in Fig. 3, the thin area is most likely a set of 4 nanosheets stacked turbostratically in *z*-direction on top of each other. Brighter spots in that image indicate the location of Ca atoms and pale spots reveal where octahedra (with Nb and O atoms sitting at the centre and on the corners, respectively) are positioned.

The atomic position of O, Nb and Ca atoms marked with colored spheres as well as the octahedra are shown on the simulated images (Fig. 6). There is no hint for the Pr atoms in the HRTEM images which might be due to the very low concentration of the dopant and the fact that different individual atoms in a regular lattice cannot be recognised by HRTEM.

**Figure 7:**
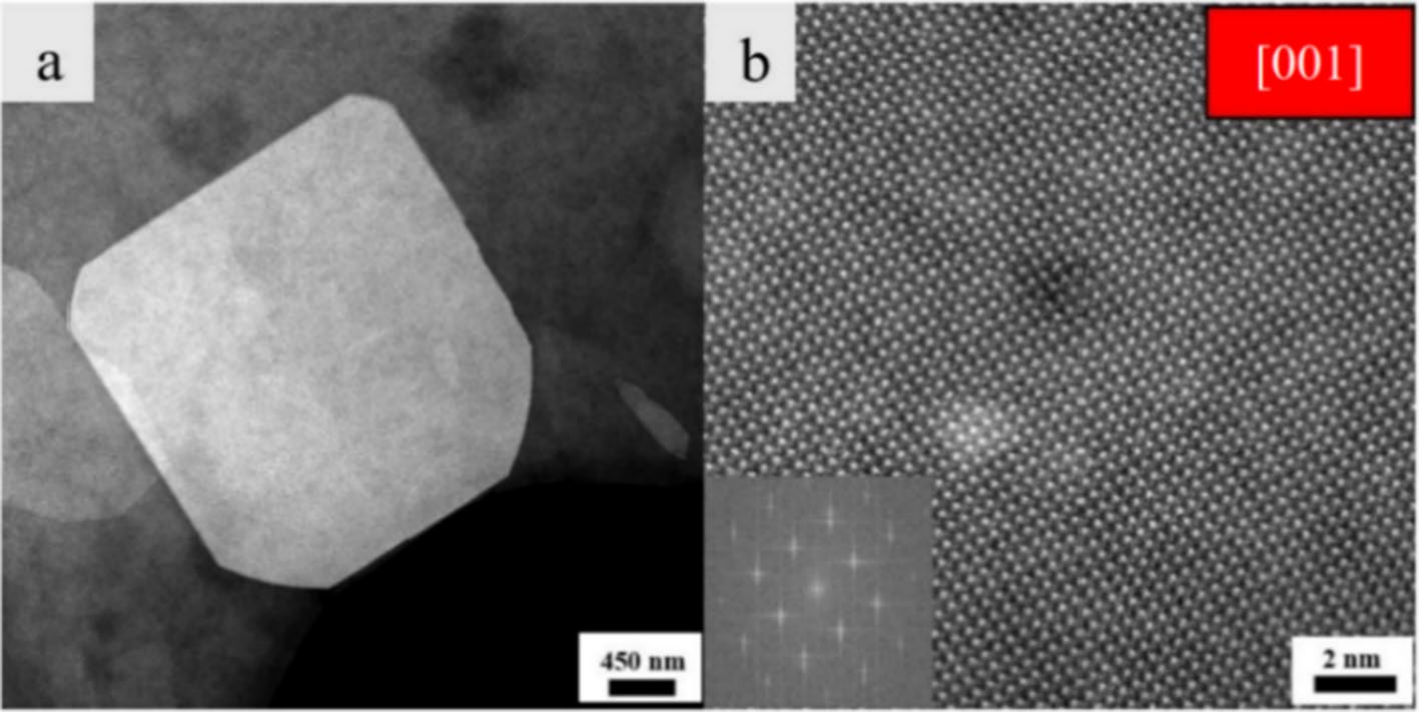
(**a**) STEM image of an individual thicker nanosheet with a high Pr content on top of the carbon support and (**b**) HRSTEM-HAADF image of the same nanosheet taken in [001]. The inset indicates the FFT pattern of the single sheet.

To be able to observe Pr atoms, STEM imaging on a thicker sheet with a high Pr content was performed (Fig. 7a). Figure 7b represents the HRSTEM of the thicker nanosheet in [001]. The inset in this image shows the FFT pattern. Since in HAADF imaging, the contrast is *Z* dependent, the atomic columns with the brightest contrast should be indicating Pr atoms’ locations. As seen on our HRSTEM image, a homogenous contrast in the atomic columns is observed. This could be due to the fact that Pr concentration is too low to be noticeably detected in addition to the fact that that STEM always gets an integral value of the whole atomic column. Only if the Pr atoms would arrange in one atomic column, they might have been visible. Thus, the fact that we cannot identify them means that they are homogenously embedded within the sheets.

### CL analysis of the nanosheets

**Figure 8:**
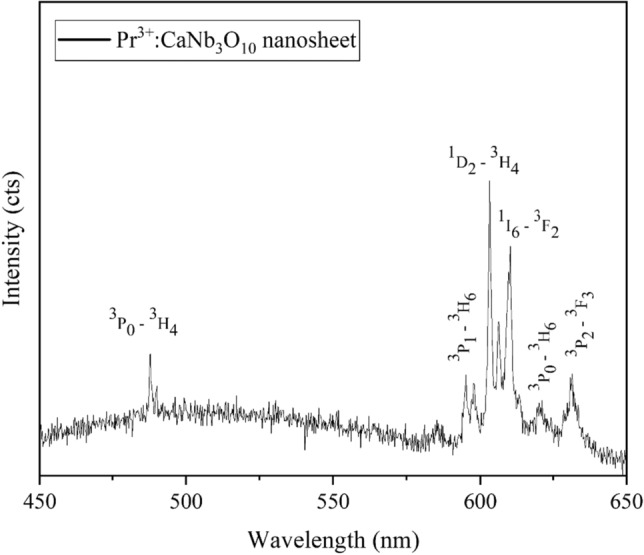
CL spectrum of an individual Pr^3+^: CaNb_3_O_10_ nanosheet. Major peaks were labelled according to the Dieke diagram^21^.

CL measurements were carried out for nanosheets with a higher Pr content. One spectrum as the representative is shown in Fig. 8. Since Pr concentration for the nanosheets is low (1.8 at%), in total CL intensity is lower compared to the PrNbO_4_ and Pr^3+^: Ca_2_Nb_2_O_7_ bulk phases which had a Pr content larger than 10 at%^10^. For reference, the CL data for the bulk from reference^10^ is given in the supplement [S2]. The positions of the emission lines are labeled. ^3^P_0_-^3^H_4_, ^3^P_0_-^3^H_6_ and ^3^P_2_-^3^F_3_ are the common transitions observed in the spectra of bulk PrNbO_4_ and Pr^3+^: Ca_2_Nb_2_O_7_. However, the ^3^P_0_-^3^H_4_ transition has a significantly lower intensity compared to these bulk phases. This transition is the dominant transition in lanthanide doped materials and can be employed for lasing activities in the material preparation^19^. Other transitions related to ^3^P_1_-^3^H_6_ (597 nm), ^1^D_2_-^3^H_4_ (603 nm) and ^1^I_6_-^3^F_2_ (610 nm) are observed only in the spectra of the nanosheets. These transitions are close to the orange light emission in the visible spectrum (600 nm), therefore, they can be used in applications where orange emission is needed. According to^20^, the emission from the ^1^D_2_ level is greatly dependent on the interaction between two nearby Pr^3+^ ions. They showed that by increasing Pr concentration, the peak related to ^1^D_2_ level disappeared in their PL data. When the Pr^3+^ concentration increases, the distance between Pr^3+^ ions decreases; as a result, concentration quenching becomes more frequent. Our result confirms this because only in the case of nanosheets with averaged Pr concentration of 1.8 at%, the transition belonging to ^1^D_2_-^3^H_4_ was detected. The mentioned transition was absent in the CL data of PrNbO_4_ and Pr^3+^: Ca_2_Nb_2_O_7_^10^.

## Conclusion

Pr^3+^-doped Ca_2_Nb_3_O_10_ nanosheets synthesized with two different Pr contents were studied. Nanosheets synthesized with a higher Pr content had the highest Pr concentration (1.8 at%), whereas for the sheets with the lower Pr content, this amount was only 0.9 at%. The structure of the nanosheets was investigated by HRTEM and HRSTEM. It was found out that the nanosheets have the same structure as the monoclinic KCa_2_Nb_3_O_10_ bulk precursor phase but with only three layers of connected Nb–O octahedra with Ca ions located in between and the K completely removed. Different thicknesses for the sheets were observed, the lowest in the range of 3 nm (corresponding to a single sheet when taking TBA^+^ molecules on top and bottom of the nanosheet into account), others up to 12 nm. The latter indicates that after exfoliation, some of the layers of the bulk structure remained stacked, leading also to a FFT pattern with only one set of reflections. Considering the presence of TBA^+^ molecules, at least 4 layers formed the thicker nanosheets as exemplarily shown with the help of multislice image simulations. CL analysis was done only on the nanosheets with the higher Pr content. Compared to the PrNbO_4_ and Pr^3+^: Ca_2_Nb_2_O_7_ bulk phases^10^, additional peaks close to 600 nm region of the visible spectrum were observed.

On the basis of these results and our previous publications^10,11^, we conclude that bulk particles (PrNbO_4_ and Pr^3+^: Ca_2_Nb_2_O_7_) have brighter emission lines (especially transitions belonging to the dominant peaks: ^3^P_0_-^3^H_4_ and ^3^P_2_-^3^F_4_) due to their much higher Pr content. However, 2D nanosheets (Pr^3+^: CaNb_3_O_10_) with additional sharp emission lines around 600 nm and smaller size are potential candidates to be used in other applications where the mentioned wavelength (orange color) is needed.

## Supplementary Information

Supplementary Figures.

## Data Availability

All data generated or analysed during this study are included in this published article [and its supplementary information files].
