# Supplementary material for: Combined structural analysis and cathodoluminescence investigations of single Pr3+-doped Ca2Nb3O10 nanosheets
Source: Sci Rep. 2023 May 17;13:8055. doi: 10.1038/s41598-023-35142-3 (PMC10192309; doi:10.1038/s41598-023-35142-3)
Supplement: Supplementary file 1 — Supplementary Figures. [file 41598_2023_35142_MOESM1_ESM.pdf]

SUPPLEMENT

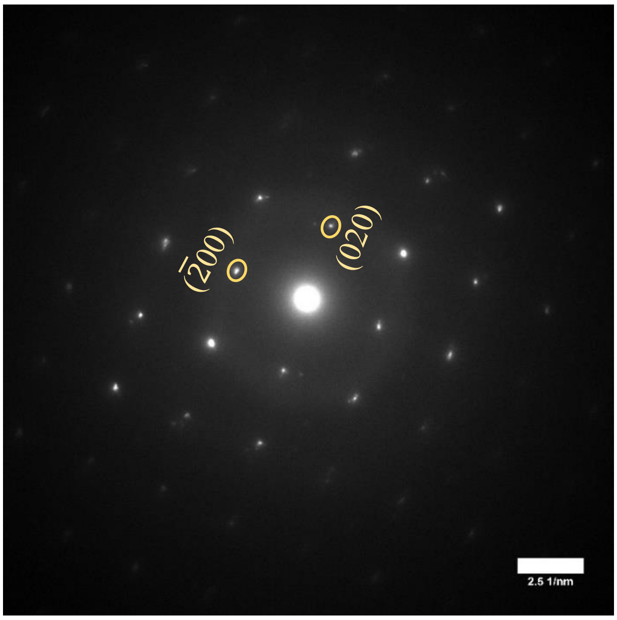

Figure S1. Selected electron diffraction pattern of a single Pr^{3+}: $Ca_2Nb_3O_{10}$ nanosheet in [001]. The pattern matches the FFT pattern shown in Fig.3 b.

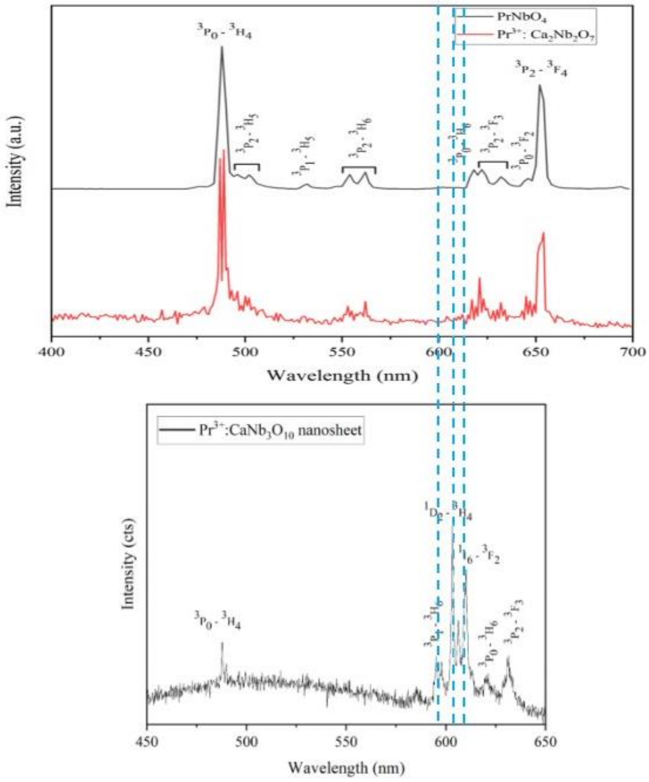

Figure S2. CL spectra of bulk particles (upper plot)[10]: $PrNbO_4$ (black curve) and Pr^{3+}:$Ca_2Nb_2O_7$ (red curve) compared with the CL spectrum of an individual nanosheet (lower plot). Additional peaks (indicated with blue lines) are only observed in the spectrum of the nanosheet.
